# REPORTING MERIT-BASED INCENTIVE PAYMENT SYSTEM LIKE DATA IN A UNIQUE AGE-FRIENDLY HEALTH CARE SETTING

**DOI:** 10.1093/geroni/igad104.1704

**Published:** 2023-12-21

**Authors:** Leland Waters, Patricia Slattum

**Affiliations:** Virginia Commonwealth University, Richmond, Virginia, United States; Virginia Commonwealth University, Richmond, Virginia, United States

## Abstract

The Richmond Health and Wellness Program (RHWP) offers screening, education, care coordination, and resources as an enhancement to primary care for older adults in five low-income apartment buildings and in a community center. Participants may receive primary care through multiple healthcare systems in the region that do not have a common electronic medical record (EMR). To track age-friendly workflows to the Health Resources Services Administration (HRSA) in the domains of advance care planning, falls prevention, and high-risk medication use, case note documentation in a REDCap database is used to generate Merit-based Incentive Payment System (MIPS)-like data. We report on advance care plans developed, fall risk screenings and risk reduction interventions implemented, high-risk medications screened, and referrals to primary care for high-risk medication deprescribing, including risk reduction with opioid use. When reporting MIPS data to HRSA, we include a disclaimer for RHWP data defining the numerator and denominator used for the MIPS-like measure calculation. For example, when reporting advance care plan completion, we include “RHWP is a care coordination site and care is not provided at the site. The population includes adults aged 55+ or people (of any age) with disabilities in low-income congregate living facilities and all adults participating in RHWP at the Virginia Commonwealth University Health Hub.” The numerator indicates the number of clients with advanced care plans created, and the denominator indicates the total number of clients seen during the reporting period. This presentation will provide strategies to meet reporting requirements when EMR records are not available.

